# Association between mistreatment of women during childbirth and symptoms suggestive of postpartum depression

**DOI:** 10.1186/s12884-022-04978-4

**Published:** 2022-08-26

**Authors:** Janini Cristina Paiz, Stela Maris de Jezus Castro, Elsa Regina Justo Giugliani, Sarah Maria dos Santos Ahne, Camila Bonalume Dall’ Aqua, Camila Giugliani

**Affiliations:** 1 Graduate Program in Epidemiology, Faculty of Medicine, Universidade Federal do Rio Grande do Sul (UFRGS), Ramiro Barcelos, 2400 – Santa Cecília, Porto Alegre, RS Brazil; 2 Department of Statistics, Universidade Federal do Rio Grande do Sul (UFRGS), Ramiro Barcelos, 2400 – Santa Cecília, Porto Alegre, RS Brazil; 3 Hospital de Clínicas de Porto Alegre, Porto Alegre, Brazil; 4 Graduate Program in Child and Adolescent Health, Faculty of Medicine, Universidade Federal do Rio Grande do Sul (UFRGS), Ramiro Barcelos, 2400 – Santa Cecília, Porto Alegre, RS Brazil; 5 Faculty of Medicine, Universidade Federal do Rio Grande do Sul (UFRGS), Ramiro Barcelos, 2400 – Santa Cecília, Porto Alegre, RS Brazil

**Keywords:** Mistreatment of women during childbirth, Obstetric violence, Postpartum depression, Childbirth, Obstetric care

## Abstract

**Background:**

Postpartum depression is a common condition in the pregnancy and postpartum cycle. The development of this condition is multifactorial and can be influenced by previous traumas. This study sought to verify whether there is an association between having been exposed to mistreatment during childbirth and presenting symptoms suggestive of postpartum depression.

**Methods:**

This is a cross-sectional study, with the inclusion of 287 women without complications in childbirth, randomly selected from two maternity hospitals of Porto Alegre, southern Brazil, in 2016. Four weeks after delivery, the postpartum women answered a face-to-face interview about socioeconomic aspects, obstetric history, health history, and childbirth experience (practices and interventions applied) and completed the Edinburgh Postnatal Depression Scale (EPDS). From the perception of women regarding the practices performed in the context of childbirth care, a composite variable was created, using item response theory, to measure the level of mistreatment during childbirth. The items that made up this variable were: absence of a companion during delivery, feeling insecure and not welcome, lack of privacy, lack of skin-to-skin contact after delivery, not having understood the information shared with them, and not having felt comfortable to ask questions and make decisions about their care. To define symptoms suggestive of postpartum depression, reflecting on increased probability of this condition, the EPDS score was set at ≥ 8. Poisson Regression with robust variance estimation was used for modeling.

**Results:**

Women who experienced mistreatment during childbirth had a higher prevalence of symptoms suggestive of postpartum depression (PR 1.55 95% CI 1.07–2.25), as well as those with a history of mental health problems (PR 1.69 95% CI 1.16–2.47), while higher socioeconomic status (A and B) had an inverse association (PR 0.53 95% CI 0.33–0.83).

**Conclusions:**

Symptoms suggestive of postpartum depression seem to be more prevalent in women who have suffered mistreatment during childbirth, of low socioeconomic status, and with a history of mental health problems. Thus, qualifying care for women during pregnancy, childbirth and postpartum and reducing social inequalities are challenges to be faced in order to eliminate mistreatment during childbirth and reduce the occurrence of postpartum depression.

## Background

Postpartum depression (PPD) is a psychiatric disorder, characterized by a predominance of depressed mood, associated with sleep disorders, feelings of worthlessness and guilt, excessive worry, difficulty concentrating, weight changes, among other symptoms, which in extreme cases can lead women to suicidal thoughts [1]. These symptoms can appear during the gestational period, soon after birth, or up to one year after delivery. PPD causes personal suffering and social dysfunction, in addition to interfering in the mother's relationship with the child [2–4].

According to the World Health Organization [5], about 10% of pregnant women and 13% of postpartum women worldwide suffer from some mental disorder, depression being the main one. In developing countries, this prevalence is 15.6% and 19.8% for pregnant and postpartum women, respectively. The different forms and methods used for diagnosis, and the characteristics of the populations studied, interfere in the estimation of the prevalence of PPD [5].

In clinical practice, screening is ideally done by asking two questions that investigate whether, in the last month, the woman has been troubled feeling depressed or hopeless; or has had little interest or pleasure in doing daily tasks [4]. If the answer is affirmative for either of the two questions, screening for PPD is recommended by applying the Edinburgh Postnatal Depression Scale – EPDS [6]. This scale is able to identify symptoms that may be associated with depressive conditions. However, the diagnosis of PPD depends on further medical evaluation to define the diagnosis [7].

PPD has a multifactorial etiology. Among the agents involved in its causality are the rapid drops in hormone levels, previous negative life events, individual susceptibilities to the development of depressive conditions, low levels of social support, marital instability, domestic violence, as well as work overload, changes in routine and sleep patterns, and the feeling of disability involved in the postpartum period [8, 9].

Recent researches investigate the association between mistreatment, disrespect and abuse in childbirth care—situations defined as obstetric violence—with the development of symptoms and presentation of PPD [10, 11]. The term obstetric violence is more commonly used in Latin America to refer to mistreatment during childbirth, and carries with it a political stance, which implies the recognition of structural problems in its origin [12]. However, in this paper we opted to use the term mistreatment because of its prevailing use in international scientific literature and also because the actual question made to the women participating in this study (see Materials and methods) contains the term mistreatment, and not the term obstetric violence [13].

Obstetric violence is a public health problem, experienced worldwide by many women, and can be defined as a violation of human rights in a period of women's vulnerability [10, 14]. Categorized as a gender-based violence, it can be expressed by verbal disrespect, physical or psychological abuse, discrimination, neglect, lack of privacy, limitation in access to information, and application of unconsented procedures [15–17].

The magnitude of mistreatment of women during childbirth varies widely, according to the different surveys conducted, with prevalence ranging from 6 to 98% [15, 18, 19]. This variation arises from the heterogeneity in the measurement of the phenomenon and recognition (or non-recognition) of the different practices performed by health professionals as abusive, disrespectful and without an evidence base for their benefits [18, 20, 21].

In the international context, studies show the existence of an association between traumatic experiences in childbirth and higher incidence of post-traumatic stress, anxiety and depression in the early (one week after birth) and late postpartum (04, 06, 12 and 24 weeks postpartum) [22–24]. In this same direction, the results of research conducted in Brazil demonstrated an association between having suffered disrespect, abuse or mistreatment during childbirth and having PPD [10, 25]. When analyzing its subcategories, significant association was found between having suffered verbal violence and developing moderate and severe PPD and having suffered physical violence and developing severe PPD [10].

The harms of PPD are not restricted to the women. Studies show that children of women with this condition are at higher risk of hospitalizations (RR 1.93; CI 95% 1.02–3.64) and mortality in the first year of life (RR 1.44; CI 95% 1.10—1.89) [26]. In the long term, these children are twice as likely to develop behavioral disorders, anxiety, depression, concentration deficits, and lower performance in school subjects such as mathematics [27, 28].

Therefore, early identification of PPD symptoms and screening of women at higher risk for this disorder is essential for diagnosis and management. Considering that mistreatment of women during childbirth seems to be a risk factor for developing PPD, it becomes relevant to further explore this association, making it possible to intervene in order to promote the women’s quality of life, including a positive motherhood experience. Thus, the aim of this study was to verify the existence of an association between mistreatment during childbirth and symptoms suggestive of PPD in a sample of women four weeks postpartum.

## Methods

### Study design, population and power calculation

A cross-sectional study, with inclusion of postpartum women who gave birth in two large maternity hospitals (one public and one private) of Porto Alegre, Rio Grande do Sul (RS), was conducted. The women were randomly selected, by drawing, in services responsible for approximately 25% of the 30,268 deliveries that occurred in the state capital city in 2016.

All women living in Porto Alegre who gave birth to full-term newborns in the two participating maternity hospitals were eligible. Women or newborns with unfavorable outcomes at delivery (death or admission to intensive care) or who presented formal contraindication for breastfeeding were excluded from the study, to avoid biases in the measurement of women’s perception of mistreatment during childbirth, postpartum depression, and other outcomes of interest in the research that originated this study, such as breastfeeding [29, 30]. Women living in areas at risk for home visits were also excluded to preserve the safety of the research team.

For the current study, the power calculation was performed prospectively, considering the sample of 287 women, to meet the objective of identifying the association between mistreatment during childbirth and symptoms suggestive of postpartum depression. The power calculated to identify an odds ratio equal to 2.5, in a model adjusted for age, skin color, education, socioeconomic status, living with a partner, parity, mental health problems, alcohol use, and pregnancy planning, considering a significance level of 5%, was equal to 85.3%. The power calculation was performed in the SAS Studio software.

### Data collection

Data collection occurred between January and August 2016. Every day, all women who had given birth in the previous 24 h and met the inclusion criteria received a number that was used for the draw. Each day, two women from the public maternity hospital and one from the private hospital were included in the study until the intended sample was reached. This proportion aimed to ensure a reasonable representation in relation to the use of public and private services, described in the literature as being around 70% and 30%, respectively, at national level [31, 32].

In the period from 31 to 37 days after delivery, an interview was conducted at the home or, rarely, in another place at the woman's preference, to apply a structured questionnaire, which was specifically designed for this study, based on the previous experience of the researchers and the guiding documents of childbirth care in Brazil [33, 34]. The questions related to mistreatment during childbirth were elaborated considering the recommendations of the WHO and the Brazilian Ministry of Health regarding good practices and a positive childbirth experience [33, 35]. Moreover, the seven dimension concepts of disrespect and abuse in facility-based childbirth, by Bowser and Hill (2010), and of mistreatment of women in childbirth at health facilities, by Bohren et al. (2015), were used as references, while considering the study context [15]. Women who were not found for the interview, after at least three attempts of telephone contact and one in person, were considered a loss.

The interviews were conducted after a pilot study that indicated the need for minor semantic adjustments to the questionnaire. The field team was composed of 12 interviewers trained for the job. Weekly meetings were held with the field team, seeking greater uniformity in data collection.

### Statistical aspects

The outcome variable of this study was symptoms suggestive of PPD. To identify these symptoms, the EPDS instrument was used, a self-administered questionnaire with 10 items, which considers the seven days preceding the interview. Each item has four possible answers, with a score associated to symptom severity. This score ranges from 0 to 3 (0: no change and; 3: significant mood change). The aspects evaluated by means of the EPDS are: ability to laugh, to find things funny, thoughts about the future, feelings (guilt, anxiety, worry, panic, overload and unhappiness), the desire to cry and to do harm to oneself [7]. The instrument has scores ranging from zero to 30, according to validation carried out in Brazil, values close to zero indicate low or no risk of PPD, while values close to or greater than 10 indicate susceptibility to PPD [36].

Considering the severity of symptoms for women and children, and the outcome of interest in this study (symptoms suggestive of PPD), and not of possible depression, which is usually based on scores between 11 and 13 [19, 36], we chose to use a score ≥ 8 points of the EPDS. This cut-off point increases the sensitivity of the instrument and allows symptomatic women, albeit with a lower score, to be identified. Previous research has already suggested that a score ≥ 8 points performs better on diagnostic tests for depressive symptoms [37].

The choice of this cut-off point is also justified by the validation of the instrument (with score ≥ 8) in the general population, in a scenario with sociodemographic, cultural, and climatic characteristics very similar to those of the present study. This study showed that the ≥ 8 cut-off point had a sensitivity of 80% and 84.4% and a specificity of 87% and 81.3%, for the general population and women, respectively [38].

The exposure of interest was mistreatment during childbirth, measured by means of a binary variable that categorized postpartum women for having or not suffered mistreatment, constructed from the latent trait Mistreatment Level of Women during Childbirth (MLWC). The original question used was: *Have you ever (during labor and childbirth care) felt disrespected, humiliated or mistreated by health professionals?* This measure, which was defined with mean zero and standard deviation 1, was based on an instrument composed of nine items calibrated by the two-parameter logistic model of Item Response Theory (IRT) [39]: not having had a companion during the prepartum, labor, and postpartum period, not having understood the information provided by professionals, not having had privacy during labor, not having felt comfortable to ask questions and participate in decisions about their care, not having felt welcomed and safe at the delivery environment, and not having had immediate skin-to-skin contact with the baby. Thirty-one variables about childbirth practices and experience were initially included in the model to construct the measure. Because they presented a significant number of missing data or did not contribute with psychometric information for the elaboration of the measure, 22 variables were removed from the model, among them: used pain relief methods, episiotomy, encouraged to walk, chose the delivery position, support for breastfeeding, among others. The cut-off point of the MLWC scale defining whether or not she had experienced mistreatment was 0.5 standard deviation above the mean. The full presentation of the development of this measure has been described in another paper [13].

Other variables were used for adjustment in this study to reduce confounding biases: age, skin color, education, socioeconomic status, living with a partner, mental health problems, alcohol use before pregnancy, parity, and if last pregnancy was planned. The socioeconomic status was assessed according to the Brazilian Research Enterprises Association [40], based on the possession of a series of domestic items and on the householder’s education level. The grouping of categories from A to E corresponds to a range from better off (A) to worse off (E). Alcohol consumption before pregnancy was measured as never, occasionally (up to twice a week) and frequent (three times or more a week) and categorized as never or any consumption (occasional or frequent).

First, relative and absolute frequencies were performed for each variable in the sample, according to the presence of symptoms suggestive of PPD. Then, crude and adjusted prevalence ratios for PPD were calculated using univariable and multivariable models, respectively. As these data are from a cross-sectional study and the objective was to estimate the magnitude of the association (prevalence ratio) between the predictor mistreatment during childbirth and the outcome symptoms suggestive of PPD, the Poisson regression model with robust variance was used for the analyses. The software used for the analyses were SAS Studio and SPSS 21.

## Results

Of the women drawn, 379 were eligible to participate in the study. Of these, 287 were interviewed. There were 25 (6.6%) refusals, and 67 (17.7%) were lost due to failure in contacting to schedule the interviews. The women not interviewed differed in terms of education and skin color, showing less education (*p* < 0.01) and a higher prevalence of white skin color compared to those interviewed (*p* = 0.032). Table 1 presents the characteristics of the women interviewed regarding sociodemographic factors, obstetric and health history, and childbirth care, and these according to the presence of symptoms suggestive of PPD.

**Table 1:** Sociodemographic, obstetric history, and childbirth care characteristics according to the frequency of symptoms of PPD

	**Sample n (%)**	**Symptoms suggestive of PPD (EPDS ≥ 8) – n (%)**	
**Predictor variables**	***n* = 287**	**Yes *n* = 82 – 28.6%**	**No *n* = 205 – 71.4%**
**Sociodemographic**			
**Age (year)**			
≤ 19 years	23 (8.0)	7 (30.4)	16 (69.6)
20–34 years	199 (69.3)	58 (29.1)	141 (70.9)
≥ 35 years	65 (22.6)	17 (26.2)	48 (73.8)
**Color of skin**			
White	216 (75.3)	66 (30.6)	150 (69.4)
Black or brown	71 (24.7)	16 (22.5)	55 (77.5)
**Socioeconomic level** (*n* = 285)			
A – B	163 (57.2)	38 (23.3)	125 (76.7)
C – D – E	122 (42.8)	44 (36.1)	78 (63.9)
**Education**			
College	124 (43.2)	34 (27.4)	90 (72.6)
Elementary and high school	163 (56.8)	48 (29.4)	115 (70.6)
**Lives with a partner**			
Yes	248 (86.4)	70 (28.2)	178 (71.8)
No	39 (13.6)	12 (30.8)	27 (69.2)
**Health status and Reproductive history**			
**Mental health problem**			
Current or past	38 (13.2)	17 (44.7)	21 (55.3)
No	249 (86.8)	65 (26.1)	184 (73.9)
**Previous births**			
One or two	240 (83.6)	72 (30.0)	168 (70.0)
Three or more	47 (16.4)	10 (21.3)	37 (78.7)
**Last pregnancy was planned**			
Yes	154 (53.7)	41 (26.6)	113 (73.4)
No	133 (46.3)	41 (30.8)	92 (69.2)
**Childbirth care**			
**Hospital status**			
Public	188 (65.5)	57 (30.3)	131 (69.7)
Private	99 (34.5)	25 (25.3)	74 (74.7)
**Had a companion**			
Prepartum	275 (95.8)	79 (28.7)	196 (71.3)
Delivery	283 (98.6)	80 (28.3)	203 (71.7)
Postpartum	275 (95.8)	78 (28.4)	197 (71.6)
**Felt comfortable asking questions** (*n* = 284)			
Yes	241 (84.9)	69 (28.6)	172 (71.4)
No	43 (15.1)	12 (27.9)	31 (72.1)
**Understood information received**			
Yes	251 (87.5)	67 (26.7)	184 (73.3)
No	36 (12.5)	15 (41.7)	21 (58.3)
**Went into labor**			
Yes	205 (71.4)	64 (31.2)	141 (68.8)
No	82 (28.6)	18 (22.0)	64 (78.0)
**Had skin-to-skin contact with the newborn** (*n* = 281)			
Yes	191 (68.0)	61 (31.9)	130 (68.1)
No	90 (32.0)	18 (20.0)	72 (80.0)
**Felt welcomed in the birth environment** (*n* = 281)			
Yes	220 (78.3)	58 (26.4)	162 (73.6)
No	61 (21.7)	23 (37.7)	38 (62.3)
**Felt safe in the birth environment** (*n* = 282)			
Yes	209 (74.1)	54 (25.8)	155 (74.2)
No	73 (25.9)	26 (35.6)	47 (64.4)
**Had privacy during birth** (*n* = 280)			
Yes	235 (83.9)	70 (29.8)	165 (70.2)
No	45 (16.1)	11 (24.4)	34 (75.6)

N other than 287 due to missing data were inserted immediately after the variable name. *EPDS* Edinburgh Postnatal Depression Scale

The sample was composed predominantly of women aged between 20 and 34 years, white, with high income and education, and who resided with their partner. Slightly more than half of the women had planned their last pregnancy. Regarding protagonism and care in childbirth, 15.1% of the women did not feel comfortable asking questions and participating in decisions, and 32% did not have skin-to-skin contact with their babies. The prevalence of symptoms suggestive of PPD, considering the cut-off point ≥ 8 was 28.6%. Table 2 shows the analysis of association of sociodemographic, health, and obstetric factors with the highest frequency of symptoms suggestive of PPD. These factors were subsequently included in the multivariable model of association between the exposure variable (mistreatment during childbirth) and the outcome.

**Table 2:** Evaluation of factors associated with symptoms suggestive of PPD in postpartum women

Factors	PR_C_ (CI 95%)	PR_A_ (CI 95%)	*P*-value*
**Age (years)**	0.99 (0.96–1.01)	1.00 (0.97–1.07)	0.857
**Color of skin**			
White	1.34 (0.83–2.16)	1.54 (0.96–2.47)	0.071
Black or brown	1.00	1.00	
**Socioeconomic level**			
A – B	**0.65 (0.45–0.93)**	**0.53 (0.33–0.83)**	**0.006**
C—D – E	1.00	1.00	
**Education**			
College (complete or incomplete)	0.92 (0.63–1.33)	1.11 (0.70–1.79)	0.648
Elementary and high school	1.00	1.00	
**Lives with partner**			
Yes	0.90 (0.54–1.49)	0.91 (0.53–1.58)	0.748
No	1.00	1.00	
**Previous births**			
One or two	0.70 (0.39–1.26)	0.64 (0.32–1.27)	0.203
Three or more	1.00	1.00	
**Planned pregnancy**			
Yes	1.16 (0.80–1.67)	1.04 (0.71–1.51)	0.841
No	1.00	1.00	
**Mental health condition**			
Yes	**1.75 (1.17–2.63)**	**1.69 (1.16–2.47)**	**0.006**
No	1.00	1.00	
**Alcohol Use**			
Yes	0.91 (0.63–1.31)	0.84 (0.57–1.25)	0.398
No	1.00	1.00	

^*^Poisson with robust variance estimation, *p*-value relative to the adjusted analysis. *PRC* Crude prevalence ratio, *PRA* Adjusted prevalence ratio

Women with a history of mental health problems had a higher prevalence of symptoms suggestive of PPD (PR 1.69; 95% CI 1.16–2.47). On the other hand, women with higher socioeconomic level had lower prevalence of these symptoms (PR 0.53; 95% CI 0.33–0.83).

Table 3 shows the association between mistreatment of women during childbirth and symptoms suggestive of PPD, with adjustment for confounding factors.

**Table 3:** Association between mistreatment of women during childbirth and symptoms suggestive of PPD

Model	PR (CI 95%)	*P*-value*
Model 1	1.58 (1.09 – 2.29)	0.016
Model 2	1.58 (1.09 – 2.30)	0.015
Model 3	1.59 (1.09 – 2.32)	0.015
Model 4	1.58 (1.08 – 2.30)	0.017
Model 5	1.49 (1.04 – 2.14)	0.029
Model 6	1.55 (1.07 – 2.25)	0.021

*PR* Prevalence ratio. * Poisson with robust variance estimation, *p*-value relative to the adjusted analysis

Model 1 = Mistreatment of women during childbirth

Model 2 = Mistreatment of women during childbirth + age

Model 3 = Model 2 + skin color + education + lives with partner

Model 4 = Model 3 + socioeconomic level

Model 5 = Model 4 + mental health condition + planned pregnancy + previous births

Model 6 = Model 5 + alcohol use

The association between mistreatment of women during childbirth and symptoms suggestive of PPD was significant both in the crude analysis (PR 1.58; 95% CI 1.09–2.29) and in the adjusted models. In Model 6, with a greater number of adjustment variables, a PR of 1.55 (95% CI 1.07–2.25) was found, with little variation in the summary measure and confidence interval after different adjustment models (Table 3).

## Discussion

Maternal depression is one of the most common mental health problems during pregnancy and childbirth, with important effects on women, children, and their families. Negative experiences related to childbirth have been associated with the occurrence of psychiatric problems, such as depression and post-traumatic stress [22, 23].

Our study identified 28.6% of women having symptoms suggestive of PPD using the cutoff point of ≥ 8 on the EPDS). We identified a significant association between mistreatment of women during childbirth and higher frequency of symptoms suggestive of PPD. Even after adjustment (for socioeconomic variables, mental health history, parity and pregnancy planning), women who suffered mistreatment during childbirth had a 50% higher prevalence of symptoms suggestive of PPD. This association possibly stems from the vulnerability related to childbirth, and the frustrations regarding the woman's expectations about the moment, the divergence between what is expected and the experience of abuse; humiliation and mistreatment trigger the trauma, which with the postpartum hormonal changes are exacerbated and favor the development of PPD.

Other studies investigating this association correlate the development of depressive symptoms with women's feelings of lack of control, not being provided with information at birth, experiencing physical pain, humiliation and abandonment, not being cared for properly and undergoing procedures without consent, as well as being frustrated with their expectations at birth and concerned about their child's health [41–43].

It is important to note that the method of mistreatment of women during childbirth adopted for this study differs from other surveys, because there is no standardized way to measure the latent variable, an issue also documented by other authors [25]. Added to this, the use of a lower cut-off point in the EPDS (≥ 8) for defining symptoms suggestive of PPD, seeking greater sensitivity, limits the comparison of this study with other surveys.

The association between having suffered mistreatment during childbirth and developing PPD was observed in other Brazilian settings [19, 25, 44]. Research conducted in the southernmost region [19] showed that having suffered verbal violence increases the chance of developing moderate PPD by more than 50% (OR = 1.58; 95% CI 1.06–2.33) and severe PPD by almost 70% (OR = 1.69; 95% CI 1.06–2.70), while having suffered physical violence more than doubles the chance of developing severe PPD (OR = 2.28; 95% CI 1.26–4.12). Another study conducted in Brazil identified a prevalence of postpartum depression of more than 50% in women who experienced physical, verbal, or negligent violence at childbirth [44].

In addition to mistreatment of women during childbirth, the present study identified low socioeconomic status and personal history of mental health problems (self-reported by the women) as factors associated with symptoms suggestive of PPD. These aspects have also been cited in other studies investigating factors associated with PPD or likely PPD conditions [10, 11]. The authors suggest that women with a history of mental health problems have specific personal characteristics that may influence their perception of the care they receive [10, 11]. Brown skin color, alcohol abuse, unplanned pregnancy, multiparity (3 or more) have been associated with likely cases of PPD in other investigations [10, 11]. Education and marital status were significant in a study assessing postpartum emotional disorders [45], but were not significant in our study.

The course of PPD is variable [46], and may have complete remission, chronicity or relapses in subsequent pregnancies—a fact that negatively impacts the quality of life of women and the mother-baby relationship, interfering in the cognitive, emotional and social development of the child [28]. These aspects should be taken into account by professionals who care for women in the pregnancy-postpartum period, in order to reduce the trivialization/normalization of this condition.

This study uses a latent variable, called mistreatment level of women during childbirth, constructed through a set of items, using IRT in modeling. The method, besides being statistically robust, evaluates each item of the measurement instrument according to its severity and discrimination capacity, allowing each one to have a different weight (importance) in estimating the mistreatment level. By using the composite variable—MLWC (mistreatment level of women during childbirth)—it is possible to measure the impacts of the absence of a companion during labor, delivery and postpartum, of the women not having felt the childbirth environment welcoming, safe and private, of not having had skin-to-skin contact with their babies immediately after childbirth, and of not having understood information and had autonomy during childbirth [13].

Another potentiality of the present study refers to the methodological rigor in its conduction, with continuous quality control and face-to-face interviews four weeks after delivery in the homes of postpartum women, aspects that increase the methodological quality, once they reduce potential biases related to the intense sensations, exhaustion and lack of time to process the facts that occurred in the immediate postpartum period and the relativization and forgetfulness related to the passing of several months after the event.

As limitations of the study, we can point out the high number of losses, with respective reduction of the sample effectively investigated, which may have hidden the association of other exposure variables with the outcome. We believe that women who lived in areas with high occurrence of violent incidents may have a lower socioeconomic status, so their exclusion from the study represents a potential selection bias. However, if we imagine that socially vulnerable women have a higher incidence of PPD [11], then the inclusion of these women would likely increase the prevalence of PPD and the magnitude of the association between PPD and MLWC. The women's previous mental health status was measured in a generic, self-reported way, without differentiating the type of psychiatric or psychological health problem, limiting a more in-depth and comparative discussion between the previous and current status. Women with negative outcomes in childbirth and mothers of babies who were admitted to the intensive care unit were not included in the study: this was a choice of the authors to reduce interference in the assessment of satisfaction with the care received, on the other hand, this may represent a potential bias, as it possibly excludes women that are less satisfied with care, victims of mistreatment and with symptoms of PPD [47].

The use of a lower cut-off point (≥ 8), less frequently used in research, limits the comparison of the findings of this study with those of other investigations and increases the proportion of false positives (1-specificity). However, in choosing this cut-off point, the highest sensitivity was valued, in search of a group of women at higher risk of developing PPD, since it is a screening and not a diagnostic tool.

## Conclusions

Women who experienced mistreatment during childbirth had a 55% higher prevalence of symptoms suggestive of PPD. The personal history of mental health problems increased by 70% this prevalence, while the higher socioeconomic status reduced the prevalence by almost 50%. Therefore, efforts to qualify childbirth care and minimize the occurrence of mistreatment, still very present in the reality of maternity hospitals worldwide, are necessary to reduce the occurrence of postpartum depression and thus prevent the various negative outcomes resulting from this condition.

The association between mistreatment during childbirth and symptoms suggestive of PPD observed in this study suggests that the use of the MLWC instrument in clinical practice may enable the identification of women susceptible to the development of PPD—a group that deserves special attention by health professionals. However, the proposed instrument needs to be validated before confirmation of this hypothesis.

## Data Availability

The datasets used and/or analyzed during the current study available from the corresponding author on reasonable request. All data generated or analyzed during this study are included in this published article.

## Abbreviations

CI: Confidence Interval
EPDS: Edinburgh Postnatal Depression Scale
IRT: Item Response Theory
MLWC: Mistreatment Level of Women during Childbirth
N: Number
OR: Odds Ratio
PPD: Postpartum Depression
PR: Prevalence Ratio
PR_A_: Adjusted Prevalence Ratio
PR_C_: Crude Prevalence Ratio
RR: Relative Risk
RS: Rio Grande do Sul
SPSS: Statistical Package for the Social Sciences
UFRGS: Federal University of Rio Grande do Sul
WHO: World Health Organization
